# Spatial-time analysis of cardiovascular emergency medical requests: enlightening policy and practice

**DOI:** 10.1186/s12889-020-10064-1

**Published:** 2021-01-04

**Authors:** Ali Azimi, Nasser Bagheri, Sayyed Mostafa Mostafavi, Mary Anne Furst, Soheil Hashtarkhani, Fateme Hashemi Amin, Saeid Eslami, Fatemeh Kiani, Reza VafaeiNezhad, Toktam Akbari, Amin Golabpour, Behzad Kiani

**Affiliations:** 1 Department of Medical Informatics, School of Medicine, Mashhad University of Medical Sciences, Mashhad, Iran; 2 Center for Mental Health Research College of Health and Medicine, Australian National University, Canberra, Australian Capital Territory Australia; 3 Center for Accident and Emergency Medicine Management, Mashhad University of Medical Sciences, Mashhad, Iran; 4 Student Research Committee, School of Health, Mashhad University of Medical Sciences, Mashhad, Iran; 5 Department of Health Information Technology, Shahroud University of Medical Sciences, Shahroud, Iran

**Keywords:** Cardiovascular disease, Emergency medical services, Spatial analysis, Geographical information systems, Ambulance services, Response time, Spatial-time

## Abstract

**Background:**

Response time to cardiovascular emergency medical requests is an important indicator in reducing cardiovascular disease (CVD) -related mortality. This study aimed to visualize the spatial-time distribution of response time, scene time, and call-to-hospital time of these emergency requests. We also identified patterns of clusters of CVD-related calls.

**Methods:**

This cross-sectional study was conducted in Mashhad, north-eastern Iran, between August 2017 and December 2019. The response time to every CVD-related emergency medical request call was computed using spatial and classical statistical analyses. The Anselin Local Moran’s *I* was performed to identify potential clusters in the patterns of CVD-related calls, response time, call-to-hospital arrival time, and scene-to-hospital arrival time at small area level (neighborhood level) in Mashhad, Iran.

**Results:**

There were 84,239 CVD-related emergency request calls, 61.64% of which resulted in the transport of patients to clinical centers by EMS, while 2.62% of callers (a total of 2218 persons) died before EMS arrival. The number of CVD-related emergency calls increased by almost 7% between 2017 and 2018, and by 19% between 2017 and 2019. The peak time for calls was between 9 p.m. and 1 a.m., and the lowest number of calls were recorded between 3 a.m. and 9 a.m. Saturday was the busiest day of the week in terms of call volume. There were statistically significant clusters in the pattern of CVD-related calls in the south-eastern region of Mashhad. Further, we found a large spatial variation in scene-to-hospital arrival time and call-to-hospital arrival time in the area under study.

**Conclusion:**

The use of geographical information systems and spatial analyses in modelling and quantifying EMS response time provides a new vein of knowledge for decision makers in emergency services management. Spatial as well as temporal clustering of EMS calls were present in the study area. The reasons for clustering of unfavorable time indices for EMS response requires further exploration. This approach enables policymakers to design tailored interventions to improve response time and reduce CVD-related mortality.

## Background

Non-communicable diseases accounted for about 73% of the total number of deaths worldwide in 2016. Of these, cardiovascular disease (CVD) was the primary cause of premature mortality, accounting for an estimated total of 9.48 million deaths worldwide, signifying a 19% increase between 2006 and 2016 [1, 2]. In Iran, there were 90,000 reported CVD-related mortalities in 2016, amounting to 25% of the total number of deaths [3]. This is despite the fact that premature CVD mortalities can often be prevented with timely delivery of emergency medical services [3]. Therefore, reducing the response time in CVD-related emergency requests is of great importance.

CVDs include ischemic heart disease or coronary artery disease (CAD) such as angina and myocardial infarction (MI) (commonly known as a heart attack), cerebrovascular disease (e.g. stroke), diseases of the aorta and arteries, including hypertension and peripheral vascular disease, congenital heart disease, rheumatic heart disease, cardiomyopathies and cardiac arrhythmias [4]. MI has the highest mortality rate compared to other conditions, and occurs as a result of a complete blockage of one of the main coronary arteries [5]. The standard clinical procedure in the treatment of MI is to restore blood flow in the blocked artery in order to avoid myocardial necrosis and restore heart function, thus reducing the risk of death. Damage to the heart muscle as a result of MI increases with time from the blockage of the artery. Interventions that facilitate the diagnosis and treatment of MI can thus potentially lead to reduced CVD-related mortalities and associated complications, and improve patients’ quality of life [6–8].

In Iran, the emergency medical services (EMS) are responsible for transferring patients to clinical centers. A patient experiencing a cardiovascular event calls 115, following which an ambulance is dispatched to the patient’s residence (scene) to assess their health status and, if deemed necessary, transfer them to the nearest health care center. Response time measures the period between a patient’s call and ambulance arrival at the scene. A quick response time is critical to enable effective delivery of clinical intervention in cardiovascular emergencies. To the best of our knowledge, no research has been conducted analyzing EMS response time for CVD incidents in Iran. Estimating and visualizing an index of EMS response time will provide essential knowledge to enable policymakers, healthcare providers and clinicians to improve response time, and ultimately reduce MI mortality rate. This response time index also needs to be adjusted to account for patients’ geographical locations and daily time periods.

Geographical information systems (GIS) is an emerging area of research increasingly being used as a decision support tool for policy makers in developing tailored interventions in healthcare [9–12]. GIS is a powerful tool to collect, manage, analyze and represent geo-referenced health data, and to identify gaps in healthcare systems [12–14]. GIS enables researchers to integrate spatial (location of health care services as geographical entities: patient locations and ambulances dispatch centers) and non-spatial data (descriptive information on geographical entities; opening hours, waiting list, etc.) into one framework to provide better informed decision making [15]. There are several international research reports on the application of GIS to the modelling of spatial accessibility and response time to EMS. The main focus points of these studies were: measuring spatio-temporal accessibility [16]; spatial variations of EMS and travel distance [17]; spatial diversity of response time for EMS [18]; the geographic-time distribution of all ambulance calls and hourly and weekly EMS call volumes [19]; and conditional autoregressive spatial models to identify drivers of cardiac arrest [20].

To our knowledge, there is no study in Iran which examines the space-time pattern of CVD-related emergency medical requests. The purpose of the present study is to perform a spatial-time analysis, using GIS, of cardiovascular emergency medical requests in Mashhad, Iran. Our specific objectives are to: 1) estimate the first index of response time for CVD-related EMS calls in Iran; and 2) investigate potential clusters in the pattern of CVD-related calls over time and space.

## Methods

### Study area and data sources

**Fig. 1:**
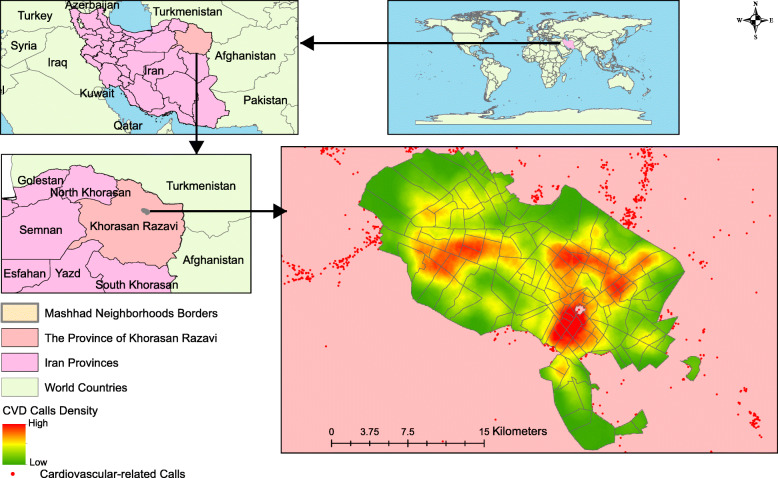
Geographical distribution of cardiovascular-related emergency calls in Mashhad, Iran. The figure created by authors

This cross-sectional study was conducted in Mashhad, north-eastern Iran, which has a population of 3,785,567 (Fig. 1). Mashhad is the most popular tourist destination and the second most populous city in Iran [21]. Mashhad has 149 neighborhoods which are divided into census blocks, the smallest unit of spatial divisions in cities of Iran. In this study, we used neighborhood catchment as the geographical scale for performing the spatial analyses. Mashhad neighborhoods have an average population of 18,523 and a mean area of 1.92 km^2^.

### Data sources

Two different data sources were used. First, data from CVD-related emergency calls were obtained from Mashhad Emergency Medical Center. The data did not contain cerebrovascular disease (e.g. stroke) incidents. Second, the spatial division of Mashhad and population data were obtained through the city municipality. The latitude and longitude for all EMS call requests were saved in the EMS database. Therefore, we did not need to geocode the data. We linked the GIS layers and EMS databases to build the geodatabase.

### Descriptive analysis

All descriptive analyses (cross tabulations and frequency indices) were performed using Excel version 2016. Descriptive maps were generated using natural break classification with five classes. Natural break classification is a data grouping method designed to determine the best arrangement of values into different classes. This is conducted by seeking to minimize each class’s average deviation from the class mean, while maximizing each class’s deviation from the means of the other groups. In other words, the method seeks to reduce the variance within classes and maximize the variance between classes [22]. ArcGIS 10.5 was used for creating the descriptive maps and spatial-time analyses.

### Cluster and outlier analysis

The incidence rate of CVD-related emergency calls was calculated using total population and number of calls in each neighborhood across the study area. The Anselin Local Moran’s *I* statistic was performed to quantify spatial autocorrelation of call frequency at the neighborhood level. This test calculates a z-score and *p*-value to determine whether the apparent similarity (a spatial clustering of either high or low values) or dissimilarity (a spatial outlier) is more pronounced than one would expect in a random distribution. The null hypothesis states that CVD-related emergency calls are randomly distributed across the study area. Areas with High-High and Low-Low clusters indicate that the target neighborhood is encompassed by neighborhoods with similar rates of CVD-related emergency calls, while High-Low and Low-High regions show that the target area is encompassed by regions with dissimilar rates of CVD-related emergency calls [9]. In other words, the High-High and Low-Low areas indicate clusters of CVD-related emergency calls occurrence, but the High-Low and Low-High areas indicate outliers of CVD-related emergency call frequencies.

### Statistical significance

Anselin local Moran’s *I* calculates a z-score and *p*-value for each feature in the dataset. *P*-value and z-score are closely associated. The p-value is the probability that the observed spatial pattern was created by some random process. A high positive z-score for a feature indicates that the surrounding features (neighboring CVD-related call values) have similar values (either high values or low values). However, a low negative z-score for a feature indicates a statistically significant spatial data outlier. Table 1 shows the range of z-scores and *p*-values used for testing the statistical significance. We used a 95% confidence level in this study, and all clusters and outliers found in this study were significant at this confidence level.

**Table 1:** Z-score and *p*-value ranges of Anselin local Moran’s *I*

Z-score (standard deviations)	*P*-value (probability)	Confidence level
< − 1.65 or > + 1.65	< 0.10	90%
< −1.96 or > + 1.96	< 0.05	95%
< −2.58 or > + 2.58	< 0.01	99%

## Results

Figure 1 shows the geographical distribution of CVD-related emergency calls in Mashhad from August 2017 until the end of 2019. There were 84,239 calls, the characteristics of which have been mapped in Table 2. It shows that only 61.64% of individuals making calls were transferred to a hospital, a figure which remained stable for each year of the study. Furthermore, on average, 2.62% of callers (2218 persons) died before ambulance arrival, 34.7% refused transfer to hospital, and for 1.04% of patients, basic services were provided by EMS technicians, along with a recommendation to personally attend a medical center for follow-up care. The mean age of men was lower than that of women (*P* < 0.001). The response time decreased from 12.33 min in 2017 to 11.07 min in 2019 (*P* < 0.001), but the time from leaving the patient’s location to arriving at hospital increased from 10.21 min in 2017 to 12.13 min in 2019 (*P* < 0.001).

**Table 2:** Characteristics of CVD-related emergency calls in the city of Mashhad from August 2017 until the end of 2019

Characteristics		All patients (*n* = 84,559)	2017 (23 Aug-30 Dec)	2018 (1 Jan- 30 Dec)	2019 (1 Jan- 30 Dec)
Mean age ± SD (*n* = 84,559)		52.83 ± 30.44	53.58 ± 21.52	52.63 ± 23.04	52.78 ± 37.64
Men mean age ± SD		51.95 ± 39.04	52.49 ± 20.81	51.72 ± 26.08	51.98 ± 50.72
Women mean age ± SD		53.60 ± 20.33	54.58 ± 22.00	53.40 ± 20.12	53.48 ± 19.99
Male (n;%)		38,998 (46.13%)	5334 (46.28%)	15,570 (45.32%)	18,094 (46.80%)
Response time	mean ± SD (*n* = 80,747)	11.35 ± 6.5	12.33 ± 5.48	11.36 ± 6.46	11.07 ± 6.77
Scene interval time	mean ± SD (*n* = 51,978)	13.02 ± 7.77	14.83 ± 11.21	12.71 ± 6.56	12.81 ± 7.53
Leave location time – hospital arrival time	mean ± SD (*n* = 42,649)	11.69 ± 8.63	10.21 ± 11.47	11.54 ± 8.19	12.13 ± 8.17
Number of call (monthly Average)		84,239 (2977)	11,533 (2685)	34,361 (2863)	38,328 (3194)
Action Result (*n* = 84,239; %)					
Transport to medical center		52,124 (61.64%)	6883 (59.68%)	21,626 (62.94%)	23,615 (61.08%)
Lack of patient cooperation and refused transfer to hospital		29,339 (34.70%)	4171 (36.17%)	11,719 (34.11%)	13,449 (34.78%)
The patient died before the ambulance arrived		2218 (2.62%)	320 (2.77%)	797 (2.32%)	1101 (2.85%)
Basic services provided by emergency technicians with patients recommended to attend a medical center		878 (1.04%)	159 (1.38%)	219 (0.64%)	500 (1.29%)

Response time or “Call receipt time – arrival time”: time interval between the time when the call is made and the time when the emergency ambulance reaches the patient [23]; Scene interval time: the time duration of the presence of emergency technician at the scene [23]; (Leave location time – hospital arrival time): The time period between ambulances leaves patient location to the hospital [23]

**Fig. 2:**
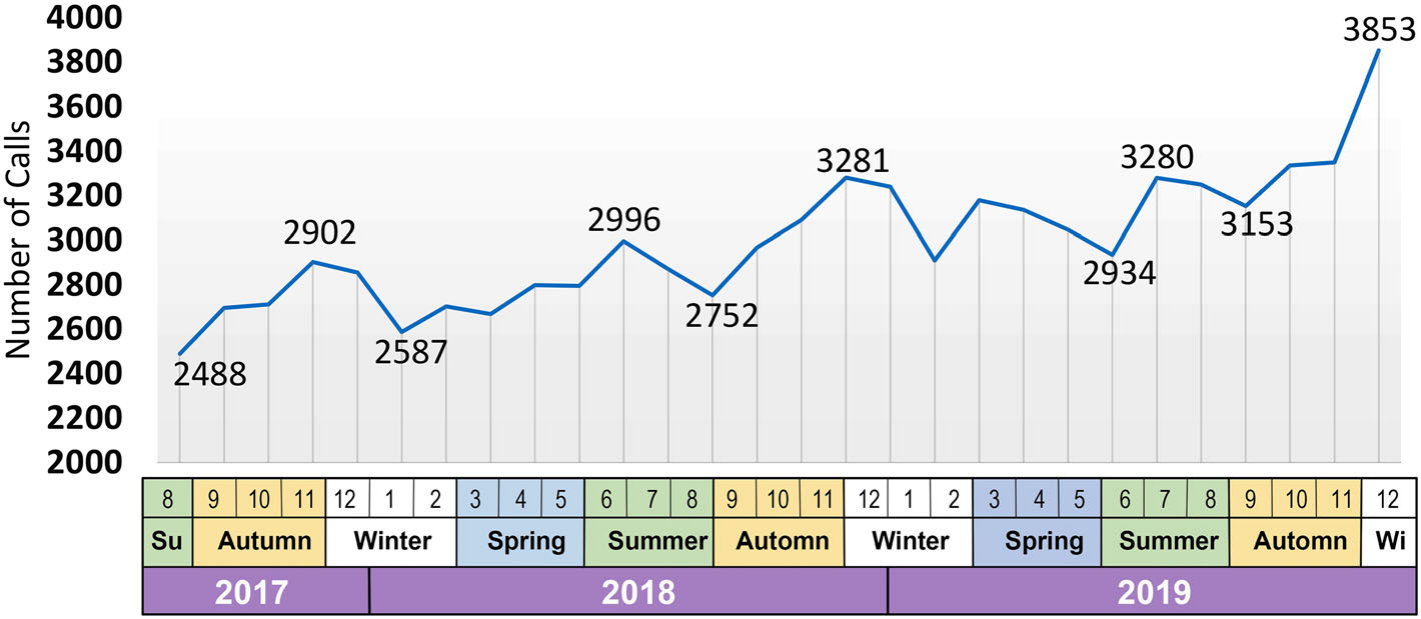
Monthly frequency of CVD-related emergency calls in the city of Mashhad, Iran during 2017–2019

Figure 2 shows the monthly temporal trend of CVD-related emergency calls in the study area between 2017 and 2019. It indicates that the number of CVD-related emergency calls increased by almost 7% between 2017 and 2018, and by 19% between 2017 and 2019. This is despite a mere 1.7% annual population growth rate in the city during this period. The figure also reveals that most CVD-related emergency calls occurred in November and December.

**Fig. 3:**
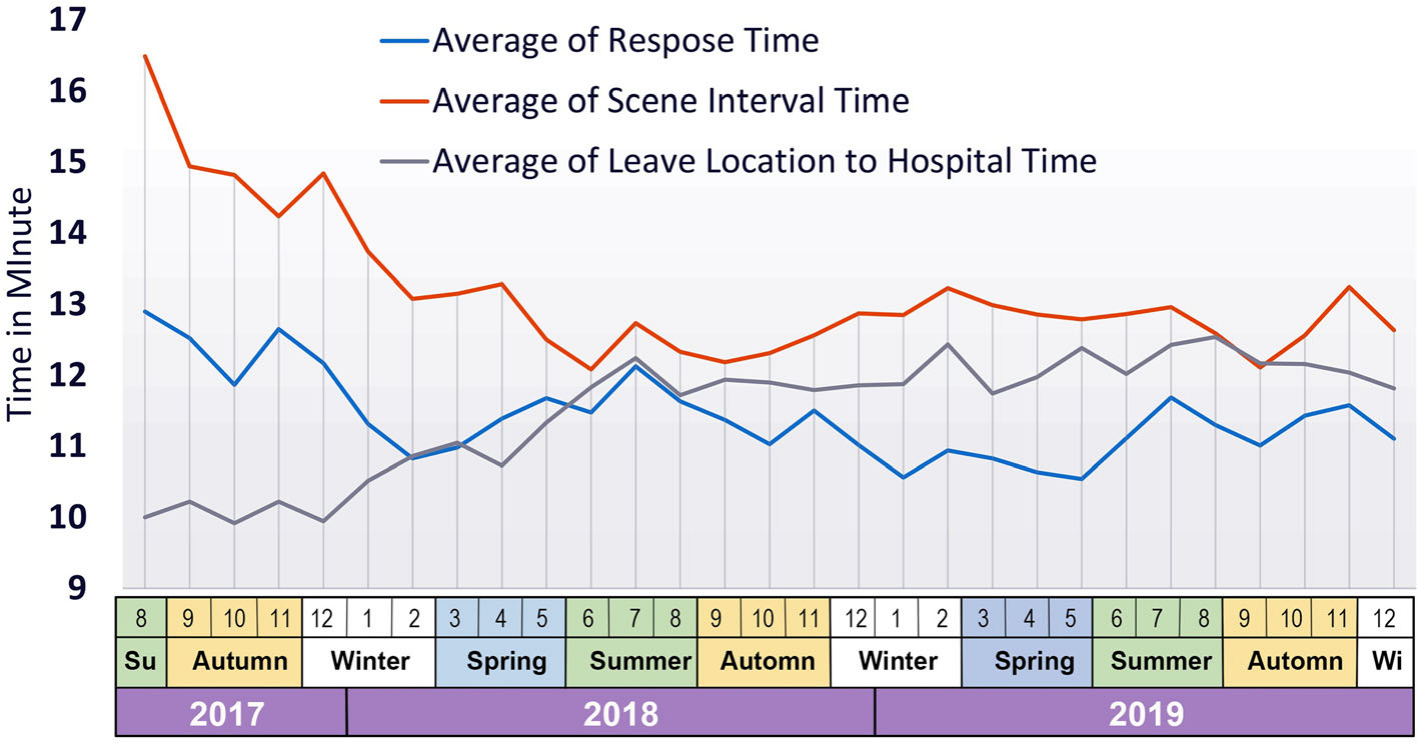
Seasonal distribution of cardiovascular-related calls in the city of Mashhad, Iran in 2017–2019

Figure 3 shows the seasonal distribution of CVD-related calls in Mashhad in 2017–2019. The highest response time was observed in the middle of summer and autumn. The figure reveals that the average response time and average scene interval time decreased, while the average time from leaving the patient’s location to arriving at hospital increased.

**Fig. 4:**
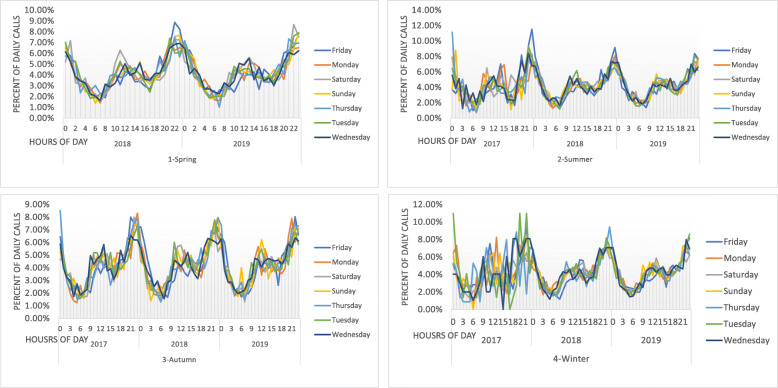
Frequency distribution of cardiovascular-related emergency calls in the city of Mashhad in 2017–2019

**Fig. 5:**
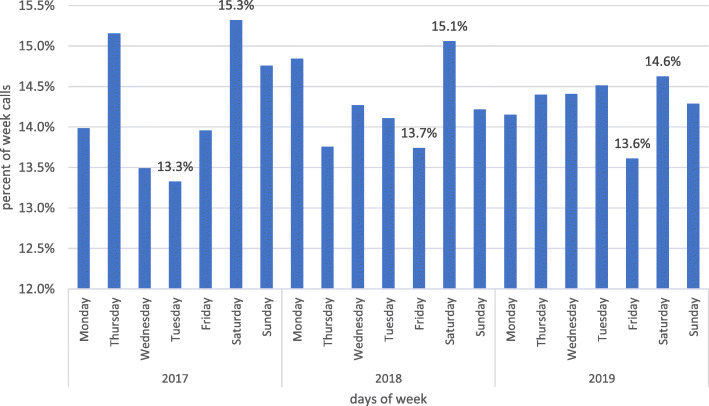
Frequency distribution of cardiovascular-related emergency calls in the days of the week in the city of Mashhad in 2017–2019

Figure 4 shows the distribution of CVD-related emergency calls according to daily time periods. The highest number of calls occurred between 9 p.m. and 1 a.m., and the lowest number of calls occurred between 3 a.m. and 9 a.m. This pattern was maintained throughout the entire period of study (2017–2019). Figure 5 shows the distribution of CVD-related emergency calls according to week days. Saturdays had the highest call volume across all 3 years. It is the first working day of the week in Iran.

**Fig. 6:**
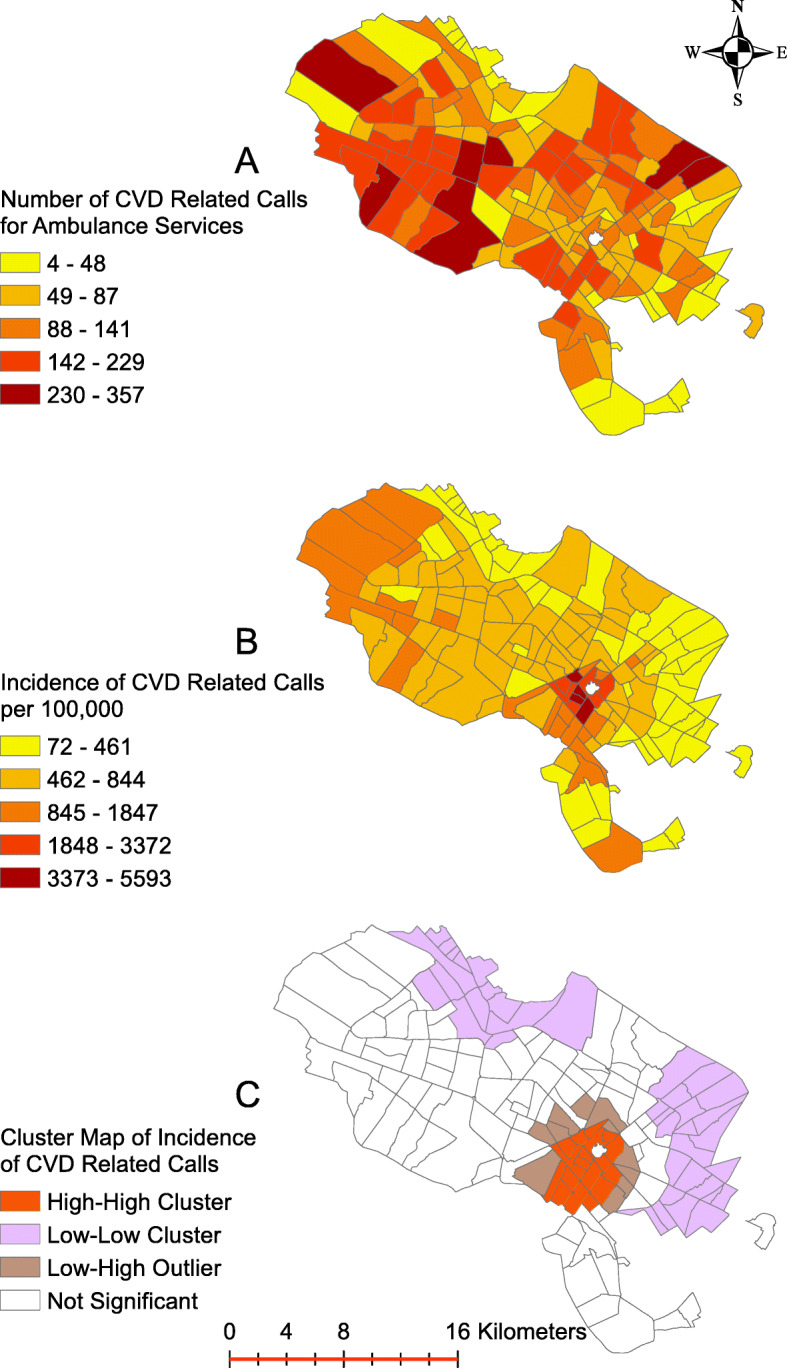
Frequency, incidence per 100,000 and incidence cluster map of cardiovascular-related emergency medical calls related to cardiovascular problems in city of Mashhad between 2017 and 2019

Figure 6 shows the geographical distribution of CVD-related emergency calls at the neighborhood level in the city of Mashhad, Iran. This graph highlights that there was a high-high cluster of the incidence of the CVD-related calls in the south-eastern area of Mashhad.

**Fig. 7:**
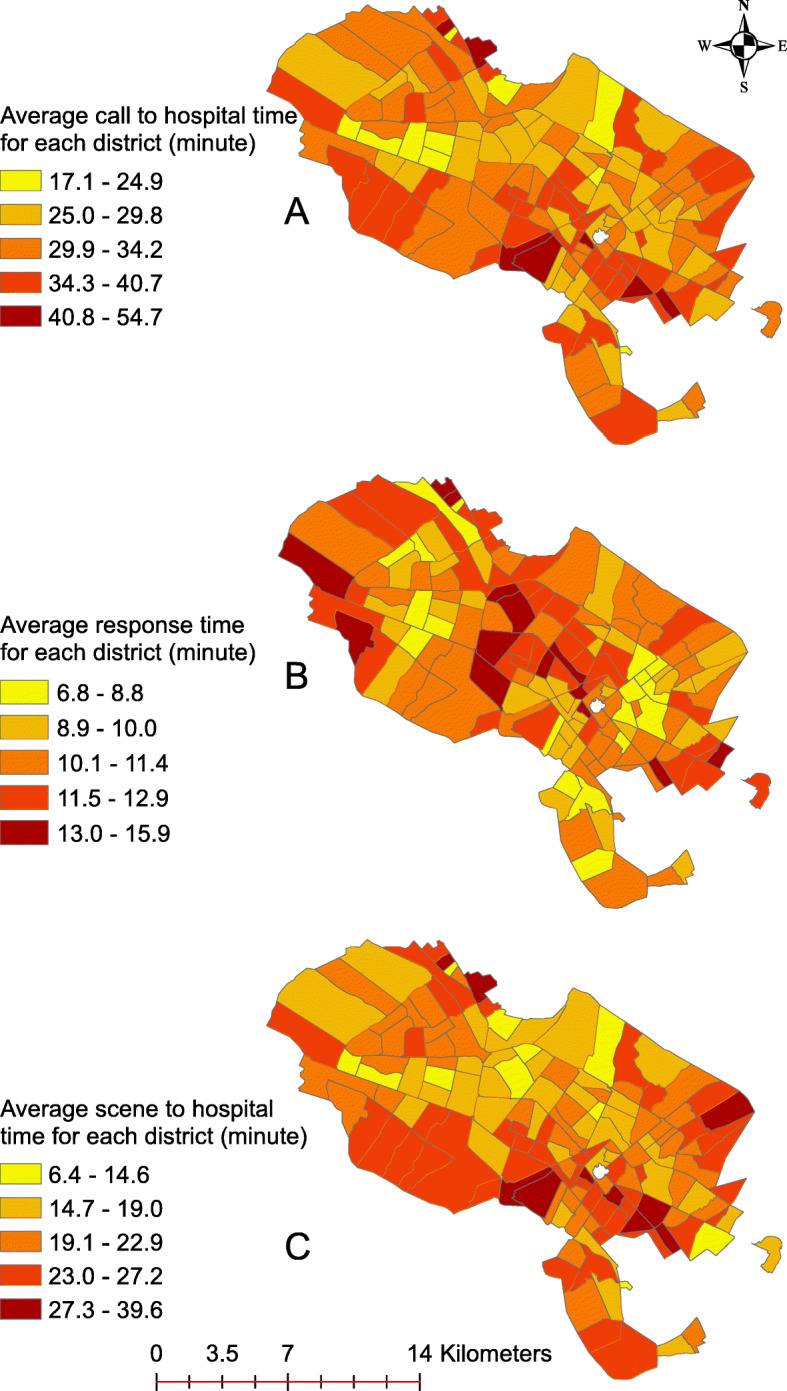
Call to hospital time, response time, and scene to hospital time map of emergency medical calls related to cardiovascular problems in city of Mashhad between 2017 and 2019

Figure 7 shows the geographical distribution of call-to-hospital time, response time, and scene-to-hospital time of emergency CVD-related medical calls in Mashhad from August 2017 until the end of 2019.

**Fig. 8:**
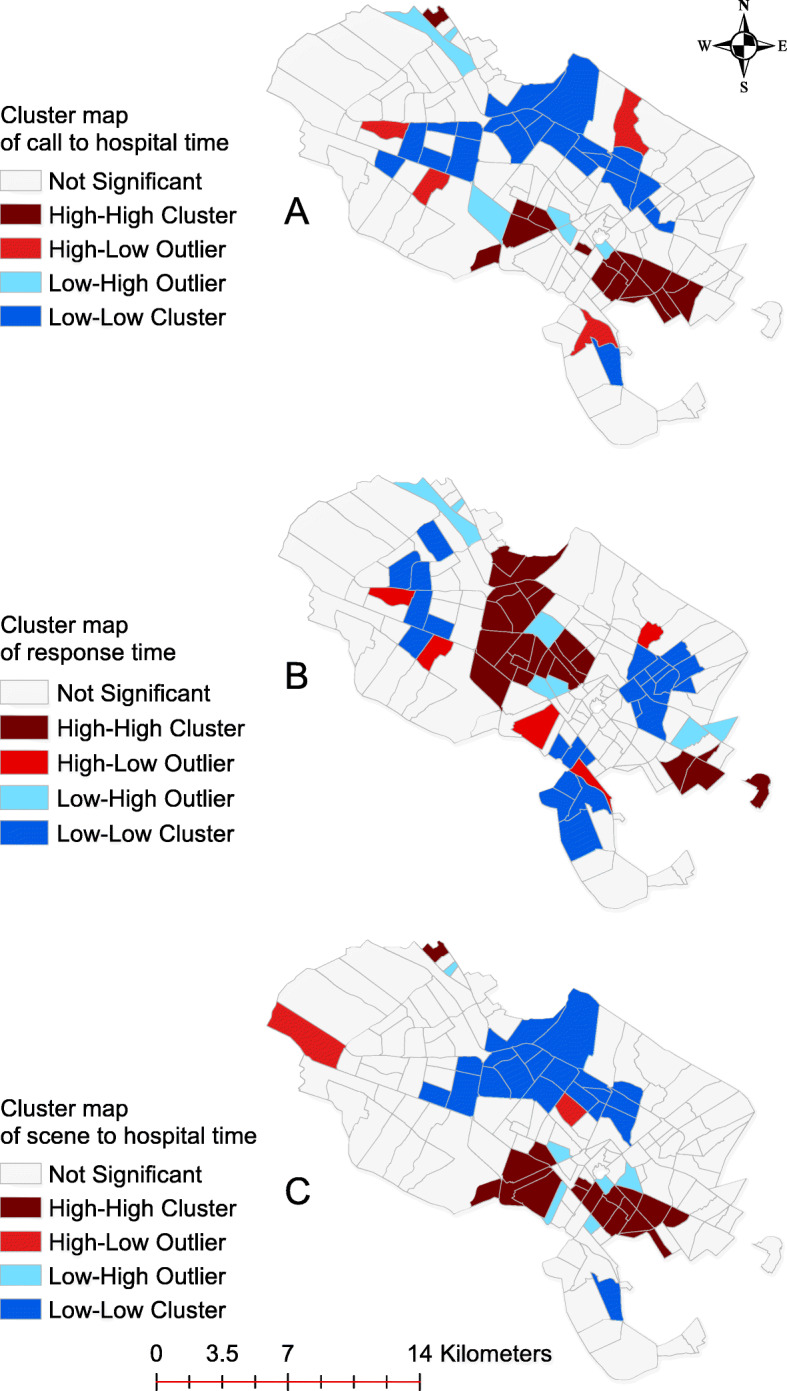
Call to hospital time, response time, and scene to hospital time cluster map of emergency medical calls related to cardiovascular problems in city of Mashhad between 2017 and 2019

Figure 8 shows that call-to-hospital-arrival time and scene-to-hospital-arrival time are the same in terms of geographical distribution at neighborhood level. However, spatial distribution of response time is different from both call-to-hospital-arrival-time and scene-to-hospital-arrival time.

**Fig. 9:**
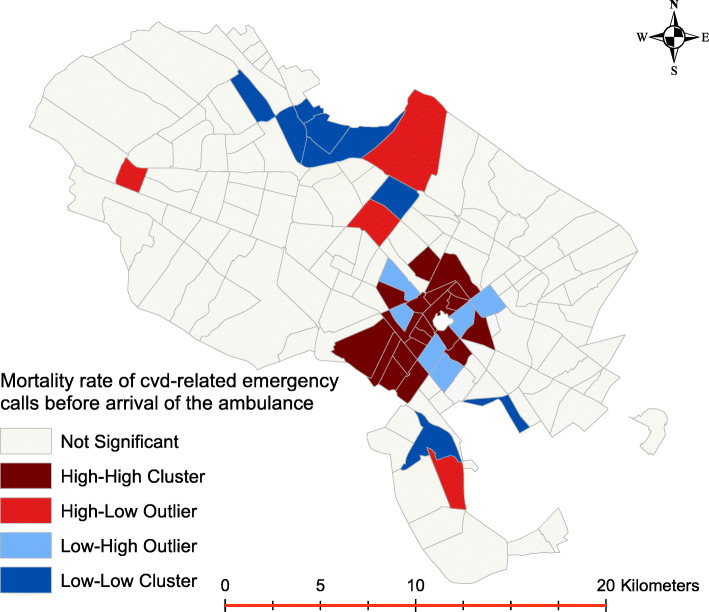
Mortality cluster map of CVD-related emergency calls before arrival of the ambulance to the patient location

Figure 9 shows the areas where CVD-related mortality is significantly higher and high-high clusters of mortality were observed in center-south part of the study area.

## Discussion

The main aim of the present study was to estimate and visualize EMS response time for CVD-related calls between 2017 and 2019. Our main findings suggest that there is a significant variation in EMS response time over space and time, with significant clusters of low/or high response time and CVD-related mortality in Mashhad.

Previous studies highlight that clinical events and calls to clinical emergency services are not random, and follow a regular pattern. This pattern depends on the specific daily time period, levels of car traffic, places of residence and commuting throughout the city, and other epidemiologic and demographic factors [19]. Sudden myocardial infarctions, for example, follow a regular pattern of occurrence [24–27], with most events occurring between morning and noon [28, 29]. Moreover, some studies report seasonal or weekly patterns of occurrence of myocardial events, with a reported increase on Mondays [30–33]. Our study findings highlighted that the number of emergency requests follows a pattern of increased incidence between morning and noon, in line with the findings of previous studies. Moreover, we also observed an increasing pattern of emergency calls between 6 pm and midnight. This pattern was observed consistently across all days of the week from 2017 to 2019 (Fig. 4). Given the fact that a previous study [34] identified sudden wake-up and heavy workload as the main risk factors for myocardial infarction, these two factors could explain the higher rate of incidence in the morning (sudden wake-up) and evening (heavy workload). Moreover, while in some studies the weekly pattern of peak incidence rate was identified as occurring on Mondays, in our study this was found to be Saturdays (Fig. 5). This is likely to be due to the difference between the working week in Iran and that of the rest of the world. In Iran, Saturday is the first day of the week, and Friday is considered to be the weekend. The lowest rate of calls was recorded on Thursdays in the first year of study, and on Fridays in the second and third year of the study. Although Fig. 2 shows that the volume of emergency services delivered increased between 2017 and 2018 by 7%, and between 2017 and 2019 by 19%, the overall ratio of cardiovascular-related emergency services to total number of emergency services delivered was 17.4, 17.5, and 17.3 for the past three calendar years, respectively. We could therefore interpret this to mean that not only CVD call requests, but all emergency calls, have increased. Furthermore, it is important to note that while these 84,329 CVD-related emergency request calls were based on symptoms interpreted by the caller as CVD related, such as chest pain, ultimately some of these calls were found not to be associated with CVD, and were instead indicators of other types of disease or disorders.

Figure 6 shows the spatial distribution of EMS calls, demonstrating that the south-eastern areas of Mashhad have the highest number of emergency service requests. This is an area in proximity to the Holy Shrine, which is a central tourist destination and a pilgrimage location. It is an area with a high concentration of hotels, residential complexes and shopping malls. As such, there is a high population density and heavy traffic congestion in this area compared to the rest of Mashhad.

The most important temporal criterion in assessing the performance of pre-hospital emergency services is response time. The standard response time is 8 min, which is directly related to higher survival rate and reduced mortality. The average response time for the three consecutive years was calculated as 11.35 ± 6.5 min, showing a pattern of decreasing response times: 12.33 ± 5.48 in 2017, 11.36 ± 6.46 in 2018 and 11.07 ± 6.77 in 2019. Another important criterion in assessing the performance of pre-hospital emergency services is scene interval, which represents the duration of time when emergency technicians are present at the scene. Patients should be managed in such a way so as to minimize delays in their transfer to clinical centers. The global gold standard for this criterion is 10 min [35]. In Iran, scene interval is typically less than 20 min [23]. The average scene interval was calculated as 13.02 ± 7.77 min for the past consecutive 3 years, showing a decreasing pattern. Although both these criteria lag far behind global standards, this difference is not uniformly distributed across different areas of the city (Figs. 7).

Cluster maps in Fig. 8 show significant differences in the performance of emergency departments across different areas of the city in relation to temporal criteria. A thorough analysis reveals that in the central areas of the city, where response time is high, there is no problem in terms of call to hospital time and scene to hospital time. The high response time may relate to a high volume of calls in this area due to its population density, leading to an increased response time in the central area of Mashhad. On the contrary, in the southern and rural areas of the city, which have a good response time, a weak call to hospital time and scene to hospital time is observed. This problem is likely due to the fact that the location of emergency dispatch centers is not properly linked with their respective hospitals. This should be taken into account to improve healthcare standards and survival rate of patients.

The cluster map of Fig. 9 shows the mortality rate of patients with myocardial infarction who died before ambulance arrival. As can be observed, the south eastern area is associated with a significantly higher risk of mortality when compared to other areas. An analysis of the mean age for these subjects indicates that they have a higher mean age (71.91 ± 20.07). This is observed across all 3 years.

Previous studies report that a higher response time is directly associated with a higher rate of mortality [36]. Although the response time is shorter in this group of patients (9.62-min vs 11.35 for the entire population), the mortality rate is higher as compared to that of the total population. This needs to be investigated further to identify other drivers (risk factors) of CVD-related mortality besides response time. Some of these risk factors have been identified in previous studies and include patients’ age, delays in requesting emergency services, previous history of disease, lack of knowledge about heart disease, and loneliness and life style [37, 38]. Given that the mean age of this group of patients is significantly higher than the rest of the population, it could be speculated that because of the higher age, other factors such as loneliness, low health literacy, previous disease history (more advanced disease, more comorbidities as a result of older age) could be the most significant driver of increased mortality despite quick response time in older individuals.

### Limitations

No data was available on the time interval between the onset of chest cardiac symptoms and the decision by the patient to request emergency services, which is an important determining factor for patient survival. Furthermore, we did not use age-standardized rates for comparison. Also, the issue of Modified Unit Area Problem remains inherent to the studies that focus on aggregated spatial datasets.

## Conclusion

The use of geographical information systems and spatial analyses in modelling and quantifying EMS response time provides a new vein of knowledge for decision makers in emergency services management. Spatial as well as temporal clustering of EMS calls were present in the study area. The reasons for clustering of unfavorable time indices for EMS response requires further exploration. This approach enables policymakers to design tailored interventions to improve response time and reduce CVD-related mortality.

## Data Availability

The datasets used and/or analyzed during the current study are available to the public from the corresponding author (B.K) on reasonable request.

## Abbreviations

CVD: Cardiovascular disease
CAD: Coronary artery disease
MI): Myocardial infarction
EMS: Emergency medical services
GIS: Geographical information systems
